# Thin bronchoscopic cryobiopsy using a nasobronchial tube

**DOI:** 10.1186/s12890-022-02166-w

**Published:** 2022-09-24

**Authors:** Masahide Oki, Hideo Saka, Yoshihito Kogure, Hideyuki Niwa, Akane Ishida, Arisa Yamada, Atsushi Torii, Chiyoe Kitagawa

**Affiliations:** 1 Department of Respiratory Medicine, National Hospital Organization Nagoya Medical Center, 4-1-1 Sannomaru, Naka-ku, Nagoya, 460-0001 Japan; 2 Department of Respiratory Medicine, Matsunami General Hospital, Gifu, Japan

**Keywords:** Bleeding, Bronchoscopy, Complications, Cryobiopsy, Thin bronchoscope

## Abstract

**Background:**

Transbronchial lung cryobiopsy is useful when diagnosing lung lesions. However, prevention of associated bleeding complications is essential. This study aimed to evaluate the safety and efficacy of our novel bronchoscopic cryobiopsy technique, which uses a long nasobronchial tube to prevent blood flooding the central airway.

**Methods:**

Patients with localized or diffuse lung lesions were prospectively enrolled and underwent cryobiopsy using a 1.9 mm diameter cryoprobe and a 4.0 mm diameter thin bronchoscope under conscious sedation. For cryobiopsy, a long silicone tube (inner diameter, 5.0 mm) was advanced through the nose to the target bronchus, then wedged to drain blood under thin-tube bronchoscopic control. The primary endpoint was the frequency of bleeding complications.

**Results:**

Of the 80 patients initially enrolled, 73 that underwent at least one cryobiopsy were ultimately included. Mild bleeding during cryobiopsy occurred in 58 patients (79.5%), but there was no moderate or severe bleeding. Other complications occurred in four patients (two pneumothorax, one pneumomediastinum, and one pneumonia). Tube dislocation was noted in eight patients (11%). Cryobiopsy specimens were significantly larger than forceps biopsy specimens (9.0 mm^2^ vs. 2.7 mm^2^, *P* < .001) and allowed specific diagnoses in 50 patients (68.5%).

**Conclusions:**

Thin bronchoscopic cryobiopsy using a nasobronchial tube in consciously sedated patients is safe and effective.

*Trial registration* Date of registration: 24/06/2019. UMIN-Clinical Trials Registry; Identifier: UMIN000037156 https://www.umin.ac.jp/ctr/index.htm

**Supplementary Information:**

The online version contains supplementary material available at 10.1186/s12890-022-02166-w.

## Background

Transbronchial cryobiopsy has become popular for the diagnosis of lung lesions, as it provides larger and higher-quality specimens than obtained with conventional forceps biopsy [1–3]. This relative ease of sampling has changed the diagnostic approach to localized lesions as well as diffuse lung diseases [3–11]. The advantage of transbronchial cryobiopsy over forceps biopsy is that larger, higher-quality specimens can be obtained for diagnostic tests. However, the potential disadvantage is a higher incidence of complications [2, 3, 12], especially bleeding. The reported rate of moderate to severe bleeding in cryobiopsy is 4.9% to 39% [2, 7, 13, 14], such that routine prophylaxis for bleeding is recommended [6, 15, 16]. Several prophylactic techniques for bleeding control, including balloon occlusion, the use of a rigid tube, and a 2-scope method, have been proposed [15–17], but their limitations include technical complexity and the need for additional instruments. We developed a novel, wedged-tube cryobiopsy technique, based on the use of a long nasobronchial tube that prevents central airway blood flooding. The technique was described in a previously published case report [18]. In the current study, we prospectively evaluated the safety and efficacy of the technique.

## Methods

### Patients

This prospective study evaluated the safety and efficacy of our novel cryobiopsy (wedged-tube) technique. From September 2019 to March 2021, patients with lung lesions were recruited and underwent cryobiopsy using the wedged-tube method [18]. The principal inclusion criterion was patients with chest-computed-tomography-confirmed lung lesions in which tissue sampling by cryobiopsy was considered adequate for a pathological diagnosis. The principal exclusion criteria were lesions inappropriate for cryobiopsy (e.g., those located within the inner second ellipse from the hilum, near a large vessel, and near large lung cysts), lesions considered to be inaccessible with a cryoprobe (left upper lobe and apical segment of the right upper lobe), and the need for bronchoscopic procedures for non-target lesions in the same setting. The study was approved by the Institutional Review Board of the National Hospital Organization Nagoya Medical Center (identifier:2019–002) and registered with the University Hospital Medical Information Network-Clinical Trials Registry (identifier: UMIN000037156, registration date: 24/06/2019). Written informed consent was obtained from all participants.

### Procedures

**Fig. 1:**
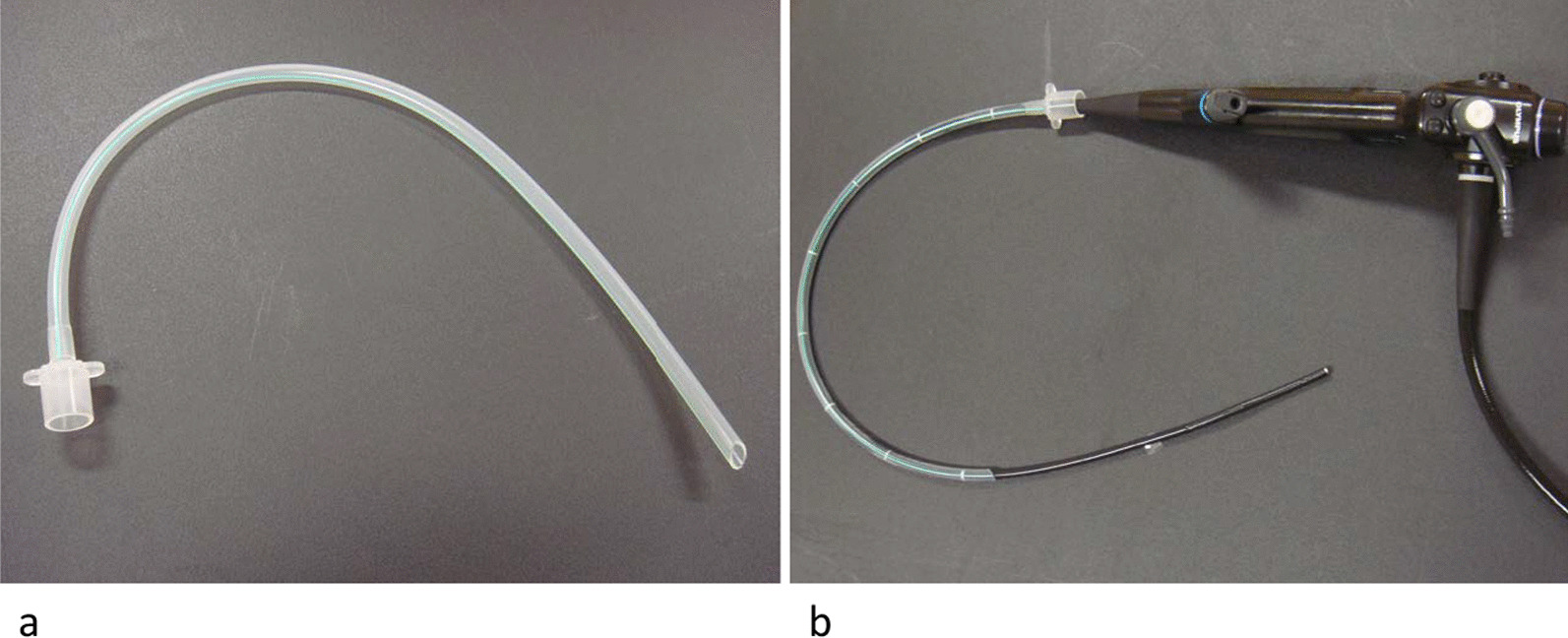
**a** A customized 5.0-mm silicone endotracheal tube with a connecter. **b** The tube was attached to a 4.0 mm bronchoscope

**Fig. 2:**
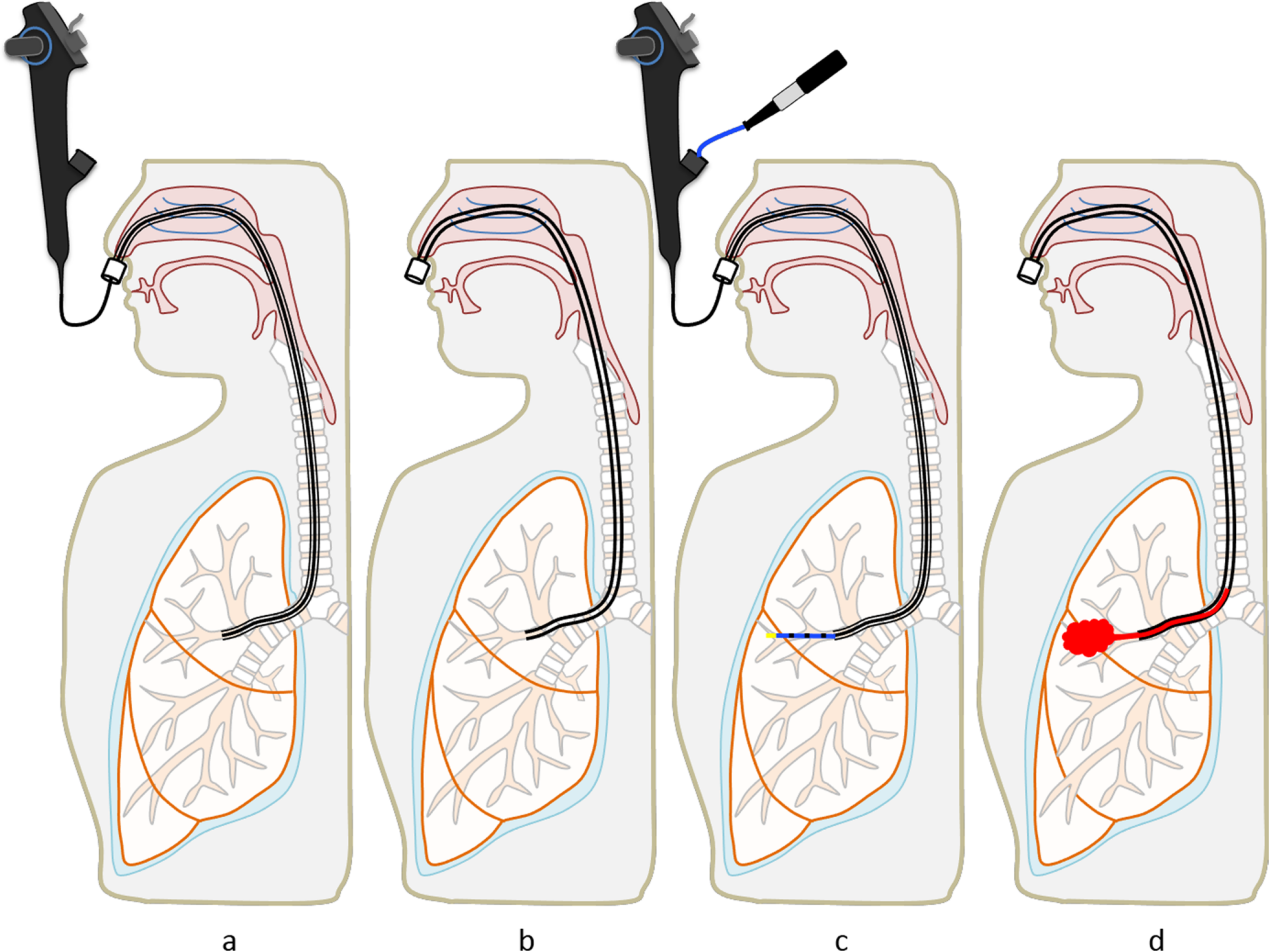
Wedged-tube cryobiopsy. **a** Advancement of the long bronchial tube through the nose to the target bronchus under thin bronchoscopic control. **b** Wedged bronchial tube. **c** Cryobiopsy. **d** Prevention of blood flooding into the central airway by draining with the bronchial tube

Bronchoscopic procedures were performed under local anesthesia with lidocaine and conscious sedation using IV midazolam and fentanyl. A sterilized, customized 5-mm inner diameter/7-mm outer diameter silicone tube (Phycon tube, SH No. 5; Fuji Systems, Tokyo, Japan; Fig. 1) was used for this procedure [18]. The silicone tube was cut to a length of 40 cm (for lesions in female patients and right upper lobe lesions) or 48 cm (for all lesions except right upper lobe lesions in male patients), and the distal tip was cut diagonally. A standard connector from a 5-mm endotracheal tube was attached to the proximal end of the tube to facilitate grasping (Fig. 1). The silicone tube was advanced through the nose to the target bronchus under bronchoscopic control using a thin bronchoscope (BF-P260F [4.0 mm outer diameter, 2.0 mm working channel, Fig. 1b] or BF-P290 [4.2 mm outer diameter, 2.0 mm working channel]; Olympus, Tokyo, Japan). We used radial endobronchial ultrasound (EBUS) and fluoroscopy in all patients to determine the biopsy site and target bronchus. After the silicone tube had been wedged firmly into the target bronchus, the thin bronchoscope was advanced beyond the tip of the tube toward the target lesion. After the target lesion had been localized with fluoroscopy and EBUS, a 1.9 mm reusable cryoprobe (ERBE, Tübingen, Germany) was advanced to the target site. Cryobiopsy was then performed with a freezing time of 5–7 s. During the procedure, an assistant held the bronchial tube firmly to prevent its migration from the wedged bronchus. The number of biopsies was left to the discretion of the operator. Before the cryobiopsy, a forceps biopsy was performed in some patients. These included patients with localized lesion into which the radial EBUS probe could not be inserted; in these cases, a forceps biopsy was done using an ultrathin bronchoscope (BF-MP290F; Olympus). Each histologic specimen was placed into a formalin-filled container, and submitted to the pathology department for interpretation. During the procedures, oxygen saturation was monitored using a pulse oximeter and supplemental oxygen (through the nasal cannula) was initiated or increased when the oxygen saturation was < 90% for 20 s. All patients rested in bed for 2 h after the procedures, and a chest radiograph was obtained routinely to identify complications. The cryobiopsy procedure using the wedged-tube method is shown in Fig. 2 and Additional file 1: Video 1.

### Outcomes

The primary outcome was the frequency of bleeding complications. Secondary endpoints were the frequency of complications other than bleeding, pathological diagnostic yield, procedural duration, frequency of oxygen desaturation, and specimen size.

Bleeding was graded as follows [15]: grade 0, no bleeding; grade 1, mild bleeding requiring bronchoscopic suctioning but not special bronchoscopic treatment; grade 2, moderate bleeding requiring bronchoscopic occlusion, bronchoscopic bronchial collapse, or cold saline instillation; grade 3, severe bleeding causing hemodynamic or respiratory instability, or requiring tamponade, surgical interventions, blood transfusion, or admission to the intensive care unit.

Specific histological findings that resulted in a definitive diagnosis, such as malignant or benign neoplasm, epithelioid cell granuloma, organizing pneumonia, and fungal infection, were considered diagnostic. Inconclusive histological findings, such as nonspecific inflammation and fibrosis, were considered non-diagnostic. Long-term follow-up and the results of multidisciplinary discussion were not used to calculate the diagnostic yield. The size of each specimen was measured using Aperio ImageScope software (Aperio Technologies Inc.), and the sizes of the largest specimens obtained by cryobiopsy and forceps biopsy in each patient were compared.

### Data analyses

The success of our technique was evaluated based on the avoidance of moderate or severe bleeding in 90% of patients. We estimated that 73 patients would be required to statistically validate the method. This number was calculated as follows: alternative completion rate of 95%, null completion rate of 80%, statistical power of 90%, and a one-sided significance level of 0.05. Eighty patients were enrolled to compensate for potential dropouts. Means and percentages are presented as appropriate. Continuous variables were compared using the Mann–Whitney *U* test. Statistical analyses were performed using PASW Statistics (ver. 18.0; SPSS Inc., Chicago, IL, USA). *P* < 0.05 was considered to indicate a statistically significant difference.

## Results

### Patients

**Fig. 3:**
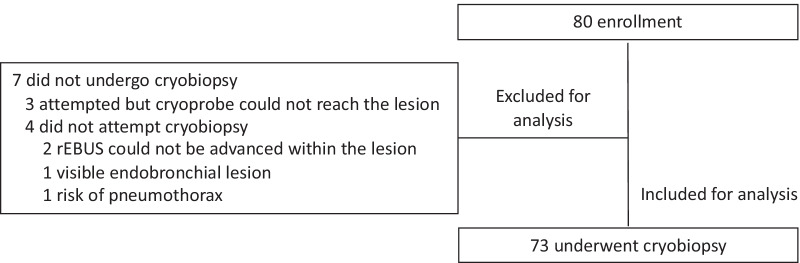
Flow chart of patient enrollment. rEBUS, radial probe endobronchial ultrasound

A total of 80 patients were enrolled, of whom 73 patients who underwent cryobiopsy were included in the analysis (Fig. 3). The baseline characteristics of the patients and lesions are listed in Table 1. Fifty-one patients (69.9%) had localized lesions and 22 patients (30.1%) had diffuse or infiltrative lesions. Bronchoscopic findings are listed in Table 2.

**Table 1:** Characteristics of the patients and their lesions

Characteristics	Value
Patients	73
Sex	
Male	45 (61.6)
Female	28 (38.4)
Age, median, years (range)	72.5 (20–88)
Smoking history	
Never	26 (35.6)
Former	31 (42.5)
Current	16 (21.9)
Target lesion	
Diffuse pulmonary diseases/lesions with infiltrative shadows	22 (30.1)
Localized lesions	51 (69.9)
Longest diameter, median, mm (range)	33.0 (11.2–78.2)
Number of bronchoscopy attempts	
First attempt	64 (87.7)
Second attempt after prior non-diagnostic bronchoscopy	9 (12.3)
Dose of sedatives	
Midazolam, mg, mean ± SD	2.7 ± 0.9
Fentanyl, μg, mean ± SD	64.5 ± 17.4

Data are presented as no. or no. (%) unless otherwise noted

**Table 2:** Histopathological findings in cryobiopsy and/or forceps biopsy specimens

Pathological findings	n (%)
Diagnostic	55 (75.3)
Localized lesions	
Adenocarcinoma	16 (21.9)
Squamous cell carcinoma	9 (12.3)
Non-small cell carcinoma	5 (6.8)
Small cell carcinoma	2 (2.7)
Diffuse large B-cell lymphoma	1 (1.4)
Granulomatous inflammation	4 (5.5)
Organizing pneumonia	3 (4.1)
Diffuse/infiltrative lung diseases	
Organizing pneumonia	5 (6.8)
Granulomatous inflammation	3 (4.1)
Adenocarcinoma	3 (4.1)
Eosinophilic pneumonia	2 (2.7)
Nonspecific interstitial pneumonia	1 (1.4)
Diffuse alveolar damage	1 (1.4)
Non-diagnostic	18 (24.7)

### Procedures

The procedural details are shown in Table 3. Of the 73 patients who underwent cryobiopsy, 59 also underwent forceps biopsies in the same setting. The silicone tube was wedged into the mean “2.5th-generation” bronchus level (range: 1st–5th; segmental bronchi were regarded as second-generation bronchi and subsegmental bronchi as third-generation bronchi). The mean number of cryobiopsy samples was 2.5 (range: 0–6). In 72 patients, at least one cryobiopsy sample was obtained. In the remaining patient, cryobiopsy was performed once and did not provide a histological specimen. This patient had a history of bronchial asthma and had a severe cough with bleeding during bronchoscopy, such that no further attempts at cryobiopsy were made. The bronchial washing revealed eosinophilic pneumonia. Cryobiopsy, forceps biopsy, and their combination provided diagnostic materials in 68.5% (50 of 73; localized, 70.6% [36 of 51]; diffuse/infiltrative, 63.6% [14 of 22]), 57.6% (34 of 59; localized, 62.0% [31 of 50]; diffuse/infiltrative, 33.3% [3 of 9]), and 75.3% (55 of 73; localized, 78.4% [40 of 51]; diffuse/infiltrative, 68.2% [15 of 22]) of the patients, respectively. The specimens obtained via cryobiopsy were significantly larger than those harvested in forceps biopsies (9.0 mm^2^ vs. 2.7 mm^2^, *P* < 0.001). The median bronchoscopy time was 33.8 min (17.0–65.3 min).

**Table 3:** Procedural details

Variables	Value
Procedures	
Cryobiopsy	73 (100)
Forceps biopsy	59 (80.8)
Only thin bronchoscopic forceps biopsy	54
Only ultrathin bronchoscopic forceps biopsy	3
Both thin and ultrathin bronchoscopic forceps biopsy	2
Number of biopsies, mean ± standard deviation (range)	
Cryobiopsy	2.5 ± 1.1 (0–6)
Forceps biopsy	7.9 ± 2.5 (3–14)
Bronchopulmonary segment for cryobiopsy	
Right upper lobe	14 (19.2)
Right middle lobe	6 (8.2)
Right lower lobe	29 (39.7)
Right upper lobe and lower lobe	1 (1.4)
Right upper lobe and middle lobe	1 (1.4)
Right middle lobe and lower lobe	2 (2.7)
Left lingula	4 (5.5)
Left lower lobe	16 (21.9)
Specific findings, n/total (%)	
Cryobiopsy	50/73 (68.5)
Forceps biopsy	34/59 (57.6)
Cryobiopsy and/or forceps biopsy, n/total (%)	55/73 (75.3)
Specimen size, median mm^2^ (range)	
Cryobiopsy, n = 72	9.0 (3.2–23.6)
Forceps biopsy, n = 59	2.7 (0.5–7.3)
Bronchoscopy time, median, min (range)	33.8 (17.0–65.3)

Data are presented as no. or no. (%) unless otherwise stated

### Complications

As shown in Table 4, bleeding after cryobiopsy occurred in 58 patients (79.5%; localized, 74.5% [38 of 51]; diffuse/infiltrative, 90.9% [20 of 22]), and all were grade 1. Five patients (localized, n = 3; diffuse/infiltrative, n = 2) had a larger amount of bleeding (> 50 mL), but in all cases it could be controlled by simple thin bronchoscopic blood suctioning within the wedged tube. No patient needed other forms of hemostatic therapy, such as cold saline/adrenaline/thrombin administration or the use of a therapeutic bronchoscope with a larger working channel. Complications other than bleeding occurred in four patients (two pneumothorax, one pneumomediastinum, one pneumonia). Pneumothoraxes and pneumomediastinum were resolved via simple observation, and pneumonia was resolved with oral antibiotics. The wedged tube was displaced in eight patients (11%), including major displacement of the tube to other lobar location in four (right middle lobe in two, lingula in two), and minor displacement to other segments in the same lobe in four (right lower lobe in two, right upper lobe in two).

**Table 4:** Safety

Variables	Value
Complications	
Bleeding	
Grade 0; no bleeding	15 (20.5)
Grade 1; mild bleeding	58 (79.5)
< 50 mL	53
> 50 mL	5
Grade 2, 3; moderate, severe bleeding	0 (0)
Pneumothorax	2^a^ (2.7)
Pneumomediastinum	1 (1.4)
Pneumonia	1 (1.4)
Supplemental oxygen during bronchoscopy	
Yes	35 (47.9)
No	38 (52.1)

Data are presented as no. or no. (%) unless otherwise stated

^a^These patients did not require thoracic drainage

## Discussion

Our study demonstrated the efficacy of the wedged-tube method in preventing blood flooding in the central airway during cryobiopsy. Moreover, this approach provides larger samples than obtained with forceps biopsy and thus contributes to a larger diagnostic bronchoscopy yield in patients with lung lesions, without compromising safety in terms of bleeding. To the best of our knowledge, this is the only transnasal cryobiopsy technique that can be performed with the patient under local anesthesia and conscious sedation.

Cryobiopsy provides larger samples than forceps biopsy, but at the expense of a higher risk of bleeding in patients with localized lung lesions or diffuse lung diseases [1–3. 10, 12]. In a randomized study of patients with diffuse lung diseases, Hetzel et al. [12] reported a significantly higher incidence of moderate to severe bleeding during cryobiopsy than during forceps biopsy (16.2% vs. 4.2%, *P* < 0.001). In a large retrospective study of 1024 patients with localized lung lesions who were placed under general anesthesia for cryobiopsy using both a rigid and a flexible bronchoscope, the frequency of moderate to severe bleeding was 8.5% [10]. In most cases, bleeding is controllable with bronchoscopy, however, near-fatal bleeding has been reported in rare cases [19, 20]. In addition, soiling of the airways with blood may exacerbate diffuse lung disease [21]. Several prophylactic methods for the control of central airway blood flooding have been proposed, including balloon blocking, use of a rigid tube, and a 2-scope method [15–17], but each has its own limitations, such as the technical difficulty, technical complexity, the need for additional instruments/equipment/medical staff, and a short but certain time lag between hemorrhage and bronchoscopic hemostasis. In the current method, the wedged tube prevents blood flooding in the central airway, by draining the blood. In fact, bleeding could be controlled with simple bronchoscopic suctioning in all patients, although the amount of bleeding differed in each one.

In the absence of a consensus definition of the bleeding grade during cryobiopsy [15, 20, 22], a comparison of our results with those of previous reports is difficult. However, our study clearly demonstrated the effectiveness of the wedged-tube method in preventing central airway blood flooding. The outer diameter of the bronchial tube used in the presence series was 7.0 mm, which is similar to the outer diameter of convex EBUS bronchoscopes. Patients can breathe spontaneously during EBUS bronchoscope procedures, as can patients who undergo wedged-tube cryobiopsy, even if the bronchial tube is filled with blood. During cryobiopsy using a balloon blocking method, the balloon may dislocate when the cryoprobe carrying the adhered frozen samples is retracted [23], possibly causing severe bleeding [20]. Inomata et al. reported an incidence of major balloon displacement, minor balloon displacement, and balloon rupture of 2.2%, 7.6%, and 1.4%, respectively [23]. Similarly, there is a risk of tube displacement in our method due to the resistance between the inner wall of the bronchial tube and the thin bronchoscope. The rate of major and minor displacements of the wedged tube was 5.5% each, which is comparable to the frequency reported by Inomata et al. Fortunately, displacement of the wedged tube did not cause bleeding complications. Although minor displacement of the tube occurred, the side wall of the tube often sealed the orifice of the target bronchus and prevented bleeding; however, major displacement can cause bleeding. To reduce this type of risk, the assistant should hold the tube firmly during the procedure. In addition, both the bronchoscope and the inner wall of the bronchial tube should be sprayed with lidocaine to facilitate the smooth removal of the bronchoscope by reducing the resistance.

The greatest advantages of the current method over other prophylactic methods are its technical simplicity and reduced invasiveness. Cryobiopsy has been performed transorally through an 8.0–9.0 mm (inner diameter) tube, using standard or large therapeutic bronchoscopes in patients under conscious sedation or general anesthesia. It has also been performed with rigid bronchoscopes in patients under general anesthesia. The current transnasal method uses a smaller-diameter bronchial tube and a thinner bronchoscope in consciously sedated patients, which is less invasive and allows the procedure to be performed on an outpatient basis. In fact, none of the 60 patients who underwent wedged-tube cryobiopsy in the outpatient setting needed unplanned hospital admission due to complications. The cryobiopsy diagnostic yields for localized lesions and diffuse lung disease range from 67 to 92% [24], and 40% to 95% [2], respectively. Many factors (e.g., size, presence of the bronchus sign, and lesion location and type) affect diagnostic yield; thus, we could not compare the diagnostic yields in the current study to published data, although ours seemed acceptable. The current technique is a good alternative to the conventional cryobiopsy technique based on the safety and efficacy profile.

Nonetheless, the wedged-tube method has a few disadvantages, especially its unavailability for the left upper lobe and right apical lung lesions. Both the left upper lobe bronchus and the right apical segmental bronchus curve sharply from the trachea, hindering advancement of the bronchial tube into those bronchi in most cases. However, the upper lobe, especially the apical segment, is rarely examined by cryobiopsy, as it is difficult to access with the stiff cryoprobe alone [25]. Another disadvantage is that larger instruments, including a 2.4 mm cryoprobe, cannot be used. However, current guidelines recommend the use of a 1.9 mm cryoprobe rather than a 2.4 mm cryoprobe in cryobiopsy [6].

Our study also had the following limitations. First, it was a single-center study conducted at an expert center, such that our results may not be generalizable. Second, long-term follow-up and the results of multidisciplinary discussions were not considered, as this study focused mainly on the safety of the wedged-tube cryobiopsy technique. The rate of false-positive results for the specific bronchoscopic findings (Table 2) was low but not zero. Third, our study excluded patients with lesions considered inappropriate for cryobiopsy or inaccessible with a cryoprobe, which may have caused selection bias. Finally, this was not a comparative study. For further insights into this technique, a large comparative study is necessary.

## Conclusions

Thin bronchoscopic cryobiopsy using a long nasobronchial tube is a safe procedure in terms of bleeding. The greatest advantage of this method over conventional cryobiopsy is that it can be performed using thinner tube, inserted transnasally in patients under conscious sedation. Because of the simplicity and reduced invasiveness, wedge-tube cryobiopsy may be a useful alternative cryobiopsy method.

## Supplementary Information

**Additional file 1: Video 1**. Cryobiopsy procedure using the wedged-tube method.

## Data Availability

The datasets used and/or analyzed during the current study are available from the corresponding author upon reasonable request.

## Abbreviation

EBUS: Endobronchial ultrasound
